# Respiratory system compliance at the same PEEP level is similar in COVID and non-COVID ARDS

**DOI:** 10.1186/s12931-022-01930-0

**Published:** 2022-01-12

**Authors:** Federica Fusina, Filippo Albani, Serena Crisci, Alessandro Morandi, Francesca Tansini, Rasula Beschi, Antonio Rosano, Giuseppe Natalini

**Affiliations:** 1 Department of Anesthesia, Intensive Care and Pain Medicine, Fondazione Poliambulanza Hospital, via Bissolati, 57, 25124 Brescia, Italy; 2 Department of Anesthesiology and Intensive Care Medicine, Catholic University of The Sacred Heart, Rome, Italy; 3 Department of Anesthesia and Intensive Care, University of Insubria, Varese, Lombardia Italy

**Keywords:** Respiratory system, Respiration, Artificial, Intensive care units, PEEP, Ventilation, Artificial, COVID-19, Respiratory system compliance

## Abstract

**Background:**

The comparison of respiratory system compliance (C_rs_) between COVID and non-COVID ARDS patients has been the object of debate, but few studies have evaluated it when considering applied positive end expiratory pressure (PEEP), which is one of the known determinants of C_rs_ itself. The aim of this study was to compare C_rs_ taking into account the applied PEEP.

**Methods:**

Two cohorts of patients were created: those with COVID-ARDS and those with non-COVID ARDS. In the whole sample the association between C_rs_ and type of ARDS at different PEEP levels was adjusted for anthropometric and clinical variables. As secondary analyses, patients were matched for predicted functional residual capacity and the same association was assessed. Moreover, the association between C_rs_ and type of ARDS was reassessed at predefined PEEP level of 0, 5, 10, and 15 cmH_2_O with a propensity score-weighted linear model.

**Results:**

367 patients were included in the study, 276 patients with COVID-ARDS and 91 with non-COVID ARDS. The association between C_rs_ and type of ARDS was not significant in both the complete cohorts (p = 0.17) and in the matched cohorts (p = 0.92). This was true also for the propensity score weighted association at PEEP 5, 10 and 15 cmH_2_O, while it was statistically significant at PEEP 0 (with a median difference of 3 ml/cmH_2_O, which in our opinion is not clinically significant).

**Conclusions:**

The compliance of the respiratory system is similar between COVID ARDS and non-COVID ARDS when calculated at the same PEEP level and while taking into account patients’ anthropometric characteristics.

**Supplementary Information:**

The online version contains supplementary material available at 10.1186/s12931-022-01930-0.

## Background

Acute respiratory distress syndrome (ARDS) is common in severe novel coronavirus 2019 disease (COVID-19) [1]. Recently, it was suggested [2] that respiratory system compliance (C_rs_) might be different in COVID-ARDS when compared to ARDS from other diseases, due to the existence of two different “phenotypes” in COVID ARDS which might not be present in non-COVID ARDS. These phenotypes have been identified as Type L, characterized by Low elastance (i.e., high compliance) and Type H, characterized by High elastance (i.e., low compliance) [2]. Subsequent studies have compared C_rs_ between COVID ARDS and non-COVID ARDS, but the debate concerning differences in C_rs_ between COVID and non-COVID ARDS patients is still ongoing, due to conflicting findings [3–6].

C_rs_ is modified by Positive End Expiratory Pressure (PEEP) levels [7–11], but previous studies have compared C_rs_ between COVID ARDS and non-COVID ARDS at different PEEP levels [3, 5, 12]. Only by knowing C_rs_ at similar PEEP levels can the C_rs_ of COVID and non-COVID ARDS patients be correctly compared, thus avoiding attributing to respiratory system characteristics what could be the effect of PEEP itself on C_rs_. Therefore, the aim of the study was to assess if C_rs_ in COVID ARDS and non-COVID ARDS is similar or different when the applied PEEP is taken into account.

## Methods

In this retrospective cohort study, data was collected from all consecutive adult subjects (over 18 years of age) with a diagnosis of ARDS (according to the Berlin Definition criteria [13]) to the Intensive Care Unit (ICU) of Poliambulanza Foundation Hospital of Brescia (Lombardy, Italy) from January 1st 2015 to May 1st 2021.The referral Ethics Committee (Comitato Etico di Brescia) approved the study (protocol number 4893).

Subjects were excluded from the analysis if: (a) they did not undergo invasive mechanical ventilation with sedation and paralysis; (b) no recordings of C_rs_ at different PEEP levels during invasive ventilation with sedation and paralysis during the ARDS period were available.

Two cohorts of subjects were created: (1) subjects with COVID-19 ARDS, i.e., the ones with ARDS attributable to Severe Acute Respiratory Syndrome–CoronaVirus- 2 (SARS-CoV -2) infection. (2) subjects with non-COVID ARDS, i.e., ARDS subjects without SARS-CoV 2 infection. ARDS patients were included in this group if ICU admission occurred before February 18th 2020, the day of the first COVID-19 diagnosis in our region (RT-PCR test for SARS-CoV-2 was not performed before this date), or if they had a negative RT-PCR test for SARS-CoV-2 after 18th February 2021.

Data on respiratory mechanics were collected, on median, on the same day as ARDS diagnosis in both COVID ARDS and non-COVID ARDS (0 [0–1] and 0 [0–1] days after ARDS diagnosis, respectively, p = 0.51). Tracheal intubation was performed, on median, on the same day as ARDS diagnosis in patients with COVID ARDS and on the day before ARDS diagnosis in patients with non-COVID ARDS (0 [0–1] and 1 [0–1.5] days before ARDS diagnosis, respectively, p < 0.001).

All measurements were taken with patients in the semirecumbent position, sedated and paralyzed. Ventilatory settings were adjusted according to our ICU’s ventilation protocol [4]: C_rs_ at different PEEP levels was assessed with a “PEEP trial”, setting volume controlled ventilation if not in use and maintaining the tidal volume set by the attending clinician in order to ensure a low-tidal volume ventilation. An end-inspiratory pause of 0.5 s was set and the respiratory rate was progressively decreased until complete exhalation was achieved. A stepwise increase (of 2 cmH_2_O) in PEEP level was performed, until an evident pattern of decrease of C_rs_ with increasing PEEP was noticeable. The lowest PEEP level at which C_rs_ was assessed in all patients was 4 cmH_2_O. A PEEP of 0 and 2 cmH_2_O could be applied in patients with SpO_2_ higher than 85%. Each PEEP level was maintained for at least 2 min [14]. When the PEEP trial was completed, the previous ventilatory mode and respiratory rate were restored, and PEEP was set to obtain a total PEEP equal to the PEEP associated with the highest C_rs_ obtained during the PEEP trial. The PEEP trial was stopped when a systematic increase in driving pressure was seen with increasing PEEP, or when adverse cardiovascular effects or a decrease in SpO_2_ greater than 10% of baseline were noticed.

The “PEEP trial” was completed, on median, on the same day as intubation in all groups.

Data (demographic, clinical, laboratory data and outcome) were extracted from the electronic medical chart of enrolled subjects.

### Measurements and calculations

Respiratory system compliance (C_rs_) was calculated as the ratio between tidal volume and driving pressure, which is the pressure distending the lungs, and is calculated as end inspiratory pressure (Plateau Pressure) minus PEEP [7].

“Best compliance” was identified as the highest C_rs_ obtained at the tested PEEP levels and “best minimum PEEP” was the lowest PEEP with which the best compliance was obtained in each patient.

Body mass index (BMI) was calculated as kg/m^2^, where kg is the weight in kilograms and m^2^ is the square of the height in meters.

Ideal Functional Residual Capacity (FRC) was calculated as 2.34·height in meters + 0.01·age in years − 1.09 in male patients and as 2.24·height in meters + 0.001·age in years − 1.00 in female patients [15]. Predicted FRC was obtained correcting for BMI with the following formula 231.9·e^(−0.070·BMI)^ + 55.2 [16]**.**

Ventilatory ratio was calculated as (tidal volume·respiratory rate·PaCO_2_)/(ideal body weight·100 ml/min·37.5 mmHg), where tidal volume is expressed in milliliters and 37.5 is assumed to be the PaCO_2_ during the ideal minute ventilation [17].

Ideal body weight (IBW) was calculated as 50 + (0.91·[height in centimeters − 152.4]) for men, and as 45.5 + (0.91·[height in centimeters − 152.4]) for women.

### Study outcomes

The main study outcome was to assess if the C_rs_ of the respiratory system was independently associated with the type of ARDS (COVID ARDS or non-COVID ARDS) when weighted for PEEP level and patients’ baseline characteristics.

### Statistical analysis

Sample size analysis was conducted with Montecarlo simulation based on data collected for a previous study [4]. Two hundred and sixty patients, hypothesising a ratio between COVID and non-COVID ARDS of 0.30, would guarantee a power of 0.80 to identify a difference in C_rs_ between the two groups of 5 ml/cmH_2_O, with a type I error frequency of 0.05.

Data are shown as count (percentage) or median (interquartile range) and comparison between non-COVID ARDS and COVID ARDS were performed with χ^2^ test and Wilcoxon rank-sum test, respectively.

We planned to analyze the association between C_rs_ and type of ARDS at different PEEP levels, using linear mixed models. The dependent variable was C_rs_ and the following covariates were chosen a priori: type of ARDS (COVID vs non-COVID), PEEP, age, sex, BMI, Ventilatory Ratio, tidal volume, PaO_2_/FIO_2_, with patients as random effect.

Two secondary analyses were planned, with the aim to repeat the analysis on matched samples: (1) two cohorts of non-COVID and COVID ARDS were created, matched for predicted FRC. Matching was performed with the nearest neighbor method. The association between C_rs_ and the type of ARDS (COVID versus non-COVID) was assessed with linear mixed models with interaction, using the type of ARDS and PEEP level at which C_rs_ was measured as covariate, and patients as random effect. (2) The average respiratory system compliance (C_rs,avg_) was calculated in each patient at a PEEP level of 0, 5, 10, and 15 cmH_2_O, taking the average C_rs_ obtained at a PEEP of 0, 5, 10 and 15 cmH_2_O ± 2 cmH_2_O. The association between C_rs,avg_ and type of ARDS (COVID versus non-COVID) was reassessed at each of the four PEEP levels with linear model weighted with stabilized Inverse Probability Treatment Weighting obtained by propensity score [18]. Propensity score was calculated using age, sex, BMI, Ventilatory Ratio, tidal volume (used in the PEEP trial) and PaO_2_/FIO_2_ as covariates.

The analyses were repeated excluding non-COVID patients with extrapulmonary ARDS.

Missing values were imputed with Multivariate Imputation by Chained Equations (Predictive Mean Matching). A p value lower than 0.05 was considered significant. Statistical analyses were performed with R 3.6.3 (R Core Team, 2021. R Foundation for Statistical Computing, Vienna) [19]**.**

## Results

**Fig. 1:**
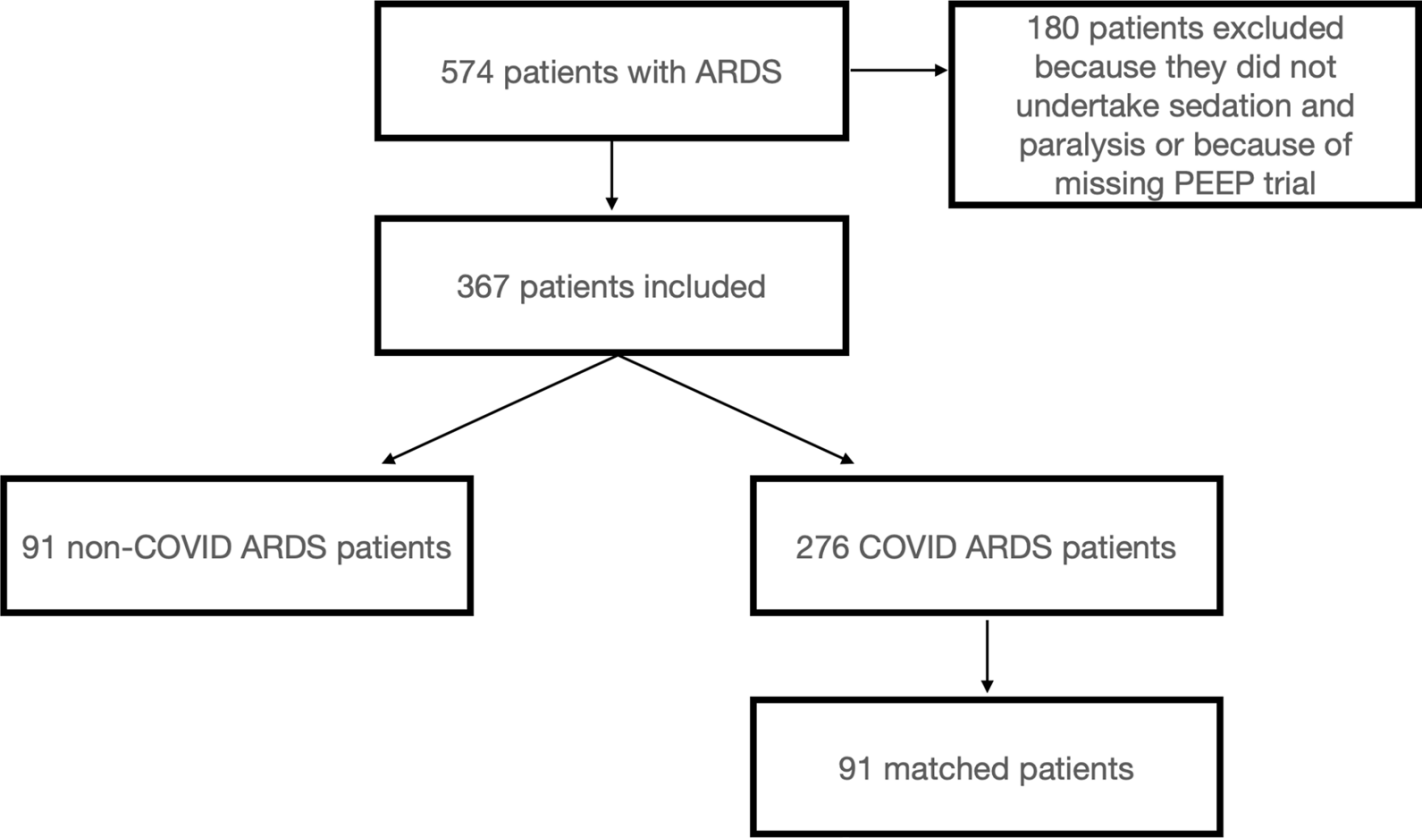
Flowchart of patients included in the study. *PEEP* positive end expiratory pressure, *ARDS* acute respiratory distress syndrome

Three hundred sixty seven subjects were included in the study, 276 with COVID-ARDS and 91 with non-COVID ARDS (Fig. 1). Patient characteristics are shown in Table 1. In 24% of non-COVID ARDS patients, the cause of ARDS was extrapulmonary. Mean arterial pressure, central venous pressure, number of patients on norepinephrine and norepinephrine dose (mcg·kg^−1^·min^−1^) and daily urine output did not differ between COVID and non-COVID ARDS patients. Heart rate and lactate had a statistically significant difference, which we believe is clinically insignificant (median heart rate 92 beats·minute [IQR 78, 105] and 83 [IQR 69, 97] in non-COVID and COVID ARDS, respectively, p = 0.001; median lactate 1.1 [IQR 0.9, 1.7] mmol·l^−1^ and 0.9 [ IQR 0.8, 1.2] mmol·l^−1^ in non-COVID and COVID ARDS, respectively, p = 0.001).

**Table 1:** Patient characteristics

	Non-COVID ARDS	COVID ARDS	p value	COVID ARDS, matched cohort	p value*
Number of patients	91	276		91	
Age, years	69 [59, 77]	68 [60, 73]	0.30	67 [63, 73]	0.40
Male sex	61 (67)	207 (75)	0.18	71 (78)	0.14
Respiratory rate, breaths·minute^−1^	21 [18, 25]	22 [20, 25]	0.16	22 [20, 24]	0.47
PEEP, cmH_2_O	8 [6, 10]	10 [8, 14]	< 0.001	10 [8, 12]	< 0.001
pH	7.34 [7.26, 7.43]	7.32 [7.23, 7.39]	0.02	7.34 [7.23, 7.39]	0.13
PaCO_2_, mmHg	44 [36, 53]	51 [44, 62]	< 0.001	49 [44, 60]	< 0.001
BMI, kg/m^2^	26 [22, 29]	28 [26, 32]	< 0.001	27 [25, 31]	0.005
V_T_, ml·kg IBW	6.4 [5.8, 7.1]	6.0 [5.4, 6.5]	< 0.001	5.9 [5.4, 6.3]	< 0.001
PaO_2_/FiO_2_	117 [89, 172]	103 [77, 138]	0.017	108 [80, 144]	0.12
Predicted FRC, liters	3.1 [2.7, 3.6]	3.1 [2.6, 3.4]	0.07	3.10 [2.7, 3.6]	0.71
Ventilatory Ratio	1.7 [1.3, 1.9]	1.9 [1.5, 2.2]	0.001	1.8 [1.5, 2.1]	0.027
Best C_rs_, ml/cmH_2_O	38 [29, 50]	40 [30, 48]	0.98	40 [30, 49]	0.82
Best minimum PEEP, cmH_2_O	6 [4, 8]	6 [2, 10]	0.64	4 [2, 8]	0.012

Data are shown as median [interquartile range], or count (percentage)

The last column (*) shows the comparison between non-COVID ARDS and COVID ARDS patients matched for predicted FRC. Best compliance is the highest C_rs_ obtained at the tested PEEP levels

Best minimum PEEP is the lowest PEEP level associated with the highest C_rs_

*PEEP* positive end expiratory pressure, *BMI* body mass index, *V*_*T*_ tidal volume, *IBW* ideal body weight, *FRC* functional residual capacity, *C*_*rs*_ respiratory system compliance, *ARDS* acute respiratory distress syndrome

**Fig. 2:**
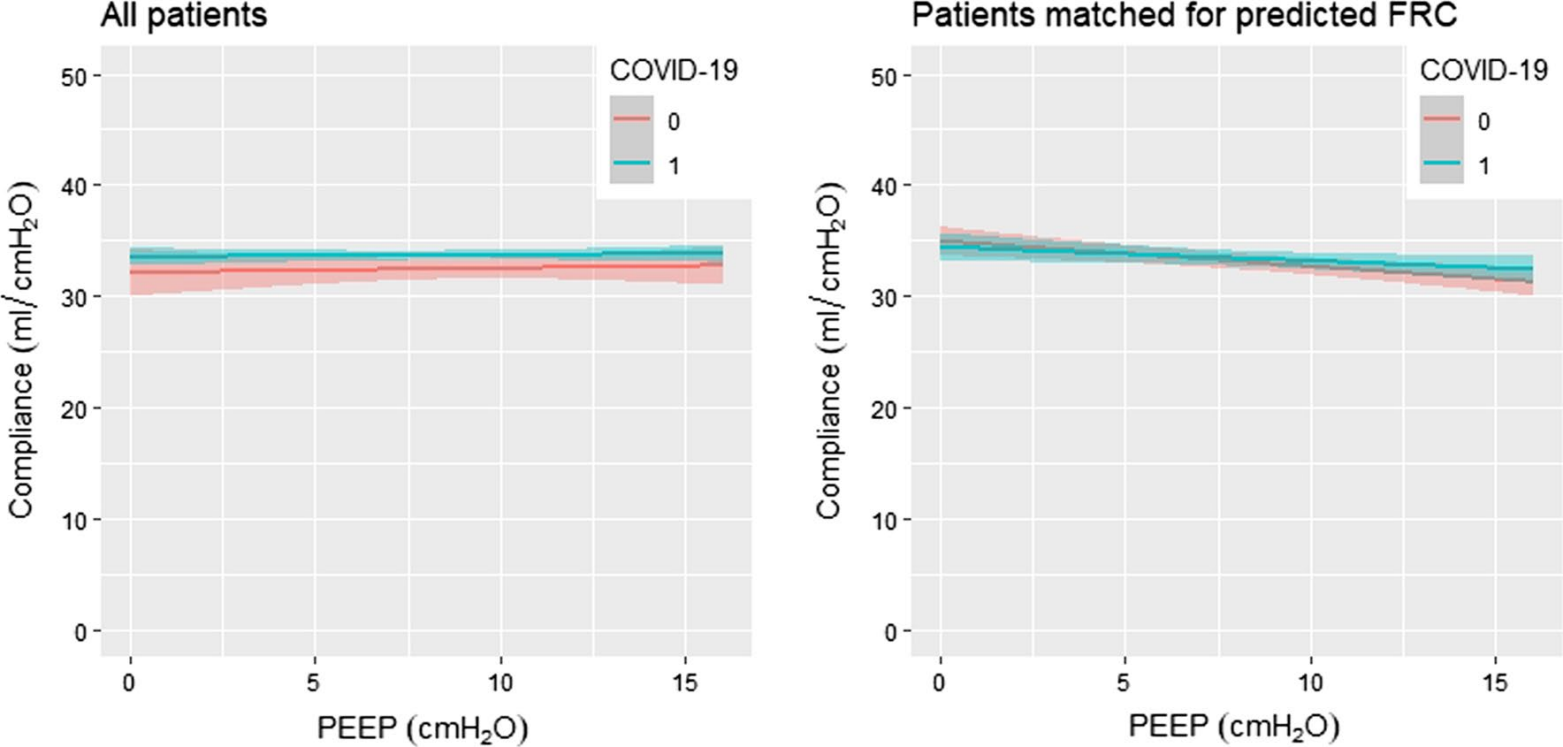
C_rs_ at different PEEP levels. Linear regression showing C_rs_ at different PEEP levels in non-COVID ARDS and COVID ARDS, in all enrolled patients and in the subset of patients which were matched for predicted FRC. 0 = non-COVID ARDS patients, 1 = COVID ARDS patients. *C*_*rs*_ respiratory system compliance, *PEEP* positive end expiratory pressure, *ARDS* acute respiratory distress syndrome, *FRC* functional residual capacity

Figure 2 shows C_rs_ at different PEEP levels in non-COVID ARDS and COVID ARDS, in all enrolled patients and in the subset of patients which were matched for predicted FRC. The association between C_rs_ and type of ARDS was not significant in both the complete cohorts (p = 0.17, see Additional file 1: Table S1) nor in the matched cohorts (p = 0.93, see Additional file 1: Table S2).

Table 2 shows the results of the propensity score weighted association between C_rs,avg_ and COVID status at different PEEP levels. C_rs,avg_ was not associated with the ARDS type at PEEP 5, 10 and 15 cmH_2_O, showing a significant association at PEEP 0, despite a clinically insignificant difference. Estimates for the other variables are displayed in Additional file 1: Table S3.

**Table 2:** Results of the propensity score weighted association between C_rs,avg_ and COVID status at different PEEP levels

	Non-COVID ARDS	COVID ARDS	p value
Number of patients	91	276	
C_rs,avg_ at PEEP 0 cmH_2_O	36 [26, 47]	33 [25, 42]	0.002
C_rs,avg_ at PEEP 5 cmH_2_O	38 [30, 48]	37 [28, 44]	0.10
C_rs,avg_ at PEEP 10 cmH_2_O	35 [28, 47]	36 [29, 44]	0.27
C_rs,avg_ at PEEP 15 cmH_2_O	32 [22, 41]	32 [24, 40]	0.13

Data are shown as median [interquartile range]

*C*_*rs,avg*_ average respiratory system compliance, *PEEP* positive end expiratory pressure, *ARDS* acute respiratory distress syndrome

**Fig. 3:**
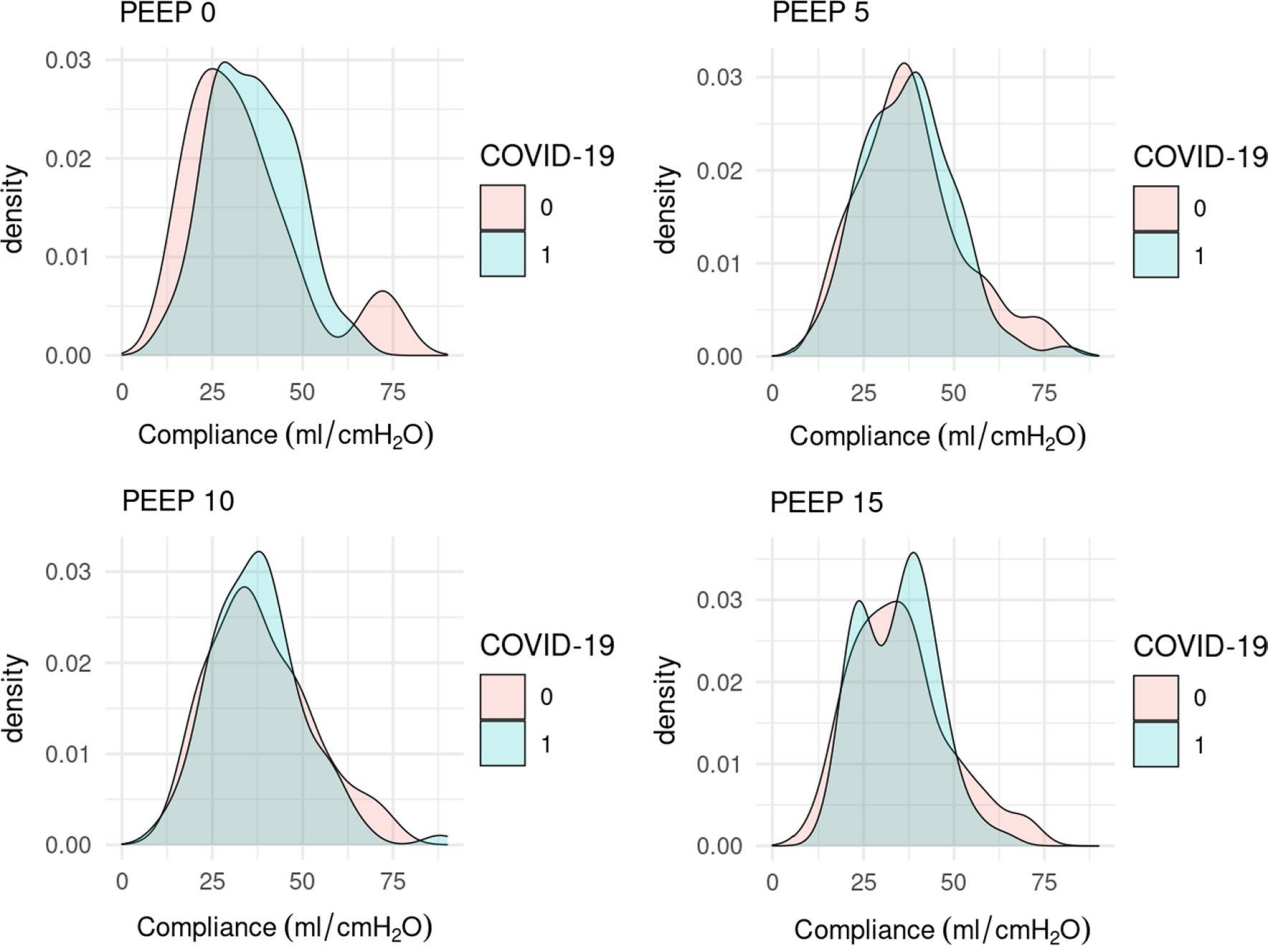
Distribution of C_rs_ at different PEEP levels. 0 = non-COVID ARDS patients, 1 = COVID ARDS patients. *C*_*rs*_ respiratory system compliance, *PEEP* positive end expiratory pressure, *ARDS* acute respiratory distress syndrome, *FRC* functional residual capacity

Figure 3 shows the distribution of C_rs,avg_ at different PEEP levels.

Results of the analyses excluding non-COVID patients with extrapulmonary ARDS are shown in Additional file 1: Tables S4–S7).

Updating the power analysis with collected data, we reached a power of 0.85 in detecting a change in compliance of 4 ml/cmH_2_O between COVID and non-COVID ARDS, with a type I error frequency of 0.05.

## Discussion

This study shows that C_rs_ is similar between COVID ARDS and non-COVID ARDS when weighted for PEEP level and patients’ baseline characteristics.

Previous studies comparing compliance between COVID-ARDS and non-COVID ARDS have shown conflicting results [3, 5, 12, 20], and the debate is still ongoing.

In particular, Haudebourg et al. [3] found similar C_rs_ between the COVID and non-COVID ARDS patients, with values close to the ones found in our study (44 ml/cmH_2_O in COVID e 42 ml/cmH_2_O in non-COVID patients in their study, versus 40 ml/cmH_2_O in COVID e 38 ml/cmH_2_O in non-COVID in our study). However, the authors did not adjust for patient characteristics, the cohorts were small (30 with COVID-ARDS versus 30 with non-COVID ARDS) and patients were not matched. In another work comparing COVID and non-COVID ARDS patients [20], the two cohorts were matched on PaO_2_/FiO_2_, FiO_2_, PEEP and tidal volume, but there was no adjustment for patient characteristics. Higher (7 ml/cmH_2_O) average values of C_rs_ were found in COVID-19 patients, but the sample size was small (30 patients) and set and total PEEP were different between COVID and non-COVID patients for both high and low PEEP levels. Other authors [5] found a higher median C_rs_ in patients with COVID-19 (41 ml/cmH_2_O) than in those with classical ARDS (32 ml/cmH_2_O). The analysis was adjusted for sequential organ failure assessment score at ICU admission, sex, age, and PaO_2_/FiO_2_ ratio, but PEEP selection was not protocolized in their patients. Ferrando et al. [12] found a tendency towards the statistical significance (p 0.06) by comparing the C_rs_ of non-COVID ARDS patients from different studies and the authors’ own data on COVID ARDS patients.

Apart from the intrinsic mechanical properties of the respiratory system, C_rs_ is affected by applied PEEP [7–11]. COVID and non-COVID patients often receive different PEEP levels during mechanical ventilation [4, 20] therefore the comparison of C_rs_ at different PEEP levels could reflect not only the impact of the disease on lung parenchyma but also the effects of the ventilatory treatment. Finding a similar C_rs_ with a different applied PEEP in COVID and non-COVID ARDS [4] does not therefore exclude that the elastic properties of the respiratory system would have been different if the same level of positive pressure had been applied. On the other hand, a different C_rs_ in COVID and non-COVID ARDS measured at a different PEEP levels cannot exclude similar mechanical properties of the respiratory system if the conditions of measurement had been the same [20].

Moreover, C_rs_ depends on the FRC: the physiological variable which links compliance to FRC is called specific compliance (the ratio between C_rs_ and FRC) [21–23]. C_rs_ is dramatically different in healthy subjects with different FRCs, ranging on average between 5 and 7 ml/cmH_2_O in newborns [20] and 120 ml/cmH_2_O in adults [21]. When C_rs_ is normalized by FRC, specific elastance (i.e., the ratio betweenFRC and C_rs_) has similar values in healthy newborns, children and adults [24]. In other words, despite similar elastic properties for each milliliter of lung tissue, the same amount of applied pressure gives a different increase in volume (and therefore of C_rs_) if the FRC is different. Matching for predicted FRC is meaningful when C_rs_ is compared in COVID and non-COVID ARDS because FRC depends on height, age, sex and BMI [15, 16, 25], and some of these characteristics have been shown to be different in COVID and non-COVID ARDS (mainly sex and BMI) [5].

The effect of a different predicted FRC could explain the findings of Li Bassi et al. [26] who found, in their analysis on COVID-19 ARDS patients, a lower C_rs_ in female patients: on average, females have a smaller stature than males, and this predicts lower FRC. Moreover, female sex by itself gives a lower predicted FRC than male sex for the same height and age, whereas BMI, which was higher for female patients in Li Bassi’s cohort, has an inverse relationship with predicted FRC. Therefore a lower C_rs_ in female patients could be better explained by different anthropometric characteristics than by a different severity of the lung disease.

Based on these pathophysiological premises and clinical data, we designed an analysis which accounted for the effect of both PEEP and FRC.

Our analyses showed that C_rs_ is not different between COVID and non-COVID ARDS if assessed at a similar PEEP level when the association was weighted for covariates, when it was carried out in a cohort matched for predicted FRC and when the inverse probability of treatment weighting was used to obtain unbiased estimates of average treatment effects. These results contribute to clarify the debate on the effect of the COVID-19 on alveolar damage, demonstrating that the inflammatory response involving the pulmonary parenchyma in ARDS affects the mechanical properties of the lung regardless of the primary disease. This is consistent with the pattern and definition of ARDS, which is considered as the common final pathway of different pulmonary and extrapulmonary diseases that spread their mediators of inflammation in the lungs [13, 27].

The similar pattern of C_rs_ in COVID and non-COVID ARDS is supported by a similar best C_rs_ obtained during the PEEP trial (median 38 versus 40 ml/cmH_2_O in non-COVID and COVID ARDS, respectively; see Table 1 for further details), and by a similar lowest PEEP at which the best C_rs_ was obtained.

As shown in Fig. 3 it is apparent that, for some patients, a high PEEP level worsens respiratory system compliance, while improving it for some patients. We can therefore speculate that the appearance of two “phenotypes” is in fact the effect of a high PEEP level, and not of COVID ARDS itself, but further studies are needed to prove these findings.

Primary analysis was also weighted on dead space, as estimated by Ventilatory Ratio [17], since one of the main differences between COVID and non-COVID ARDS appears to be the dead space ventilation, expression of altered perfusion and vascular endothelial injury [4, 5, 28]. The peculiar increase in dead space ventilation of COVID-19 has been explained as a consequence of pulmonary microvascular thrombosis which locally increases the ventilation/perfusion ratio [5, 28, 29].

The dead space increase in COVID ARDS compared with non-COVID ARDS is confirmed in our analysis, with a higher ventilatory ratio in COVID ARDS compared to non-COVID ARDS in both unmatched and matched cohorts.

This study has two main limitations. First, it is a single center study and therefore requires further confirmation. Nonetheless, since we analyzed the association of C_rs_ and type of ARDS for a wide range of PEEPs, we believe that our data could be generalized to centers which use a different approach to mechanical ventilation. Second, it is a retrospective study, even if data was prospectively collected. More research is needed on the subject in order to corroborate these findings.

## Conclusion

The compliance of the respiratory system is similar between COVID ARDS and non-COVID ARDS when calculated at the same PEEP level and while taking into account patients’ anthropometric characteristics.

## Supplementary Information

**Additional file 1.** Supplementary Material.

## Data Availability

The datasets used and/or analysed during the current study are available from the corresponding author on reasonable request.

## Abbreviations

ARDS: Acute respiratory distress syndrome
COVID-19: Novel coronavirus 2019 disease
C_rs_: Respiratory system compliance
PEEP: Positive end expiratory pressure
SARS-CoV -2: Severe Acute Respiratory Syndrome–CoronaVirus- 2
RT-PCR: Reverse transcriptase-polymerase chain reaction
ICU: Intensiva care unit
FRC: Functional residual capacity
BMI: Body mass index
IBW: Ideal body weight
C_rs,avg_: Average respiratory system compliance
