# Supplementary material for: Respiratory system compliance at the same PEEP level is similar in COVID and non-COVID ARDS
Source: Respir Res. 2022 Jan 12;23:7. doi: 10.1186/s12931-022-01930-0 (PMC8753591; doi:10.1186/s12931-022-01930-0)
Supplement: Supplementary file 1 — Additional file 1. Supplementary Material. [file 12931_2022_1930_MOESM1_ESM.docx]

**Table 1S. Association between compliance and COVID status adjusted for PEEP level and other relevant variables**

|  | beta estimate (95% CI, p value) |
| --- | --- |
| COVID ARDS | 1.893 (-0.833, 4.619, p=0.17) |
| PEEP, cmH_2_O | -0.136 (-0.177, -0.095, p<0.001) |
| Age, years | 0.044 (-0.062, 0.150, p=0.42) |
| Male sex | 12.638 (9.746, 15.531, p<0.001) |
| BMI, kg/m^2^ | -0.184 (-0.409, 0.040, p=0.11) |
| Ventilatory Ratio | -0.399 (-2.515, 1.717, p=0.71) |
| V_T_, ml⋅kg IBW | 4.362 (2.962, 5.762, p<0.001) |
| PaO_2_/FiO_2_ | 0.009 (-0.012, 0.030, p=0.41) |

A p value ≤ 0.05 was considered statistically significant.

Abbreviations: ARDS = acute respiratory distress syndrome, PEEP= positive end expiratory pressure, BMI= body mass index, V_T_,= tidal volume, IBW= ideal body weight, CI= confidence interval

**Table 2S. Association between compliance and type of ARDS (COVID or other disease) adjusted for PEEP level in non-COVID ARDS and COVID ARDS patients matched for FRC**

|  | beta estimate (95% CI, p value) |
| --- | --- |
| COVID ARDS | -0.144  (-3.130 , 2.842, p=0.93) |
| PEEP, cmH_2_O | -0.236  (-0.306 , -0.166, p<0.001) |
| Compliance PEEP interaction | 0.016  (-0.083 , 0.115, p=0.76) |

A p value ≤ 0.05 was considered statistically significant.

Abbreviations: ARDS = acute respiratory distress syndrome, PEEP= positive end expiratory pressure, CI= confidence interval

**Table 3S. Association between compliance and COVID status at four PEEP levels**

|  | PEEP 0,  beta estimate (95% CI, p value) | PEEP 5,  beta estimate (95% CI, p value) | PEEP 10,  beta estimate (95% CI, p value) | PEEP 15,  beta estimate (95% CI, p value) |
| --- | --- | --- | --- | --- |
| COVID ARDS | -4.350 (-7.012 , -1.688, p=0.002) | -2.600  (-5.697, 0.497, p=0.10) | -1.608  (-4.475 , 1.258, p=0.27) | -2.089  (-4.799, 0.621, p=0.13) |
| Age, years | 0.09  (-0.010 , 0.190, p=0.08) | 0.112  (-0.005, 0.228, p=0.06) | 0.077  (-0.031, 0.185, p =0.16) | 0.024  (-0.077 , 0.126, p =0.64) |
| Male sex | 13.473  (10.715, 10.715, p<0.001) | 15.395  (12.185, 18.604, p<0.001) | 13.579  (10.609, 16.549, p<0.001) | 12.725  (9.917, 15.533, p<0.001) |
| BMI, kg/m^2^ | -0.251  (-0.472, -0.029, p=0.028) | -0.187  (-0.445 , 0.070, p = 0.16) | -0.041  (-0.280 , 0.197, p = 0.74) | 0.222  (-0.004 , 0.447, p = 0.055) |
| Ventilatory Ratio | -1.230  (-3.417 , 0.958, p=0.27) | -0.413  (-2.959 , 2.132, p=0.75) | -0.848  (-3.204 , 1.508, p=0.48) | -0.830  (-3.058 , 1.397, p=0.47) |
| V_T_, ml⋅kg IBW | 3.656  (2.330, 4.982, p<0.001) | 4.675  (3.132, 6.219, p<0.001) | 4.597  (3.169, 6.026, p<0.001) | 3.987  (2.636,5.337, p<0.001) |
| PaO_2_/FiO_2_ | 0.014  (-0.007, 0.03, p=0.20) | 0.016  (-0.009, 0.040, p=0.22) | 0.011  (-0.012, 0.034, p=0.36) | 0.018  (-0.004 , 0.040, p=0.11) |

A p value ≤ 0.05 was considered statistically significant.

Abbreviations: PEEP= positive end expiratory pressure, ARDS = acute respiratory distress syndrome, BMI= body mass index, V_T_,= tidal volume, IBW= ideal body weight, CI= confidence interval

**Table 4S. Association between compliance and COVID status adjusted for PEEP level and other relevant variables, excluding extrapulmonary ARDS**

|  | beta estimate (95% CI, p value) |
| --- | --- |
| COVID ARDS | 1.868 (-0.975, 4.710, p=0.20) |
| PEEP, cmH_2_O | -0.126 (-0.168, -0.084, p<0.001) |
| Age, years | 0.059 (-0.053, 0.171, p=0.30) |
| Male sex | 13.252 (10.250, 16.254, p<0.001) |
| BMI, kg/m^2^ | -0.175 (-0.405, 0.055, p=0.14) |
| Ventilatory Ratio | -0.674 (-2.850, 1.502) p=0.54) |
| V_T_, ml⋅kg IBW | 4.530 (3.080, 5.980, p<0.001) |
| PaO_2_/FiO_2_ | 0.010 (-0.012, 0.032, p=0.38) |

A p value ≤ 0.05 was considered statistically significant.

Abbreviations: ARDS = acute respiratory distress syndrome, PEEP= positive end expiratory pressure, BMI= body mass index, V_T_,= tidal volume, IBW= ideal body weight, CI= confidence interval

**Table 5S. Association between compliance and type of ARDS (COVID or other disease) adjusted for PEEP level in non-COVID ARDS excluding extrapulmonary ARDS patients** **and COVID ARDS patients matched for FRC**

|  | beta estimate (95% CI, p value) |
| --- | --- |
| COVID ARDS | 0.282  (-3.002, 3.567, p=0.87) |
| PEEP, cmH_2_O | -0.223  (-0.302, -0.145, p<0.001) |
| Compliance PEEP interaction | 0.044  (-0.067, 0.155, p=0.44) |

A p value ≤ 0.05 was considered statistically significant.

Abbreviations: ARDS = acute respiratory distress syndrome, PEEP= positive end expiratory pressure, CI= confidence interval

**Table 6S. Association between compliance and COVID status at four PEEP levels, excluding extrapulmonary ARDS**

|  | PEEP 0,  beta estimate (95% CI, p value) | PEEP 5,  beta estimate (95% CI, p value) | PEEP 10,  beta estimate (95% CI, p value) | PEEP 15,  beta estimate (95% CI, p value) |
| --- | --- | --- | --- | --- |
| COVID ARDS | -4.751  (-7.528, -1.974, p=0.001) | -2.497  (-5.993, 1.000, p=0.16) | -1.852  (-4.927,1.224, p=0.24) | -2.246  (-5.158, 0.665, p=0.13) |
| Age, years | 0.066 (-0.037, 0.169, p=0.21) | 0.129  (-0.001, 0.258, p=0.052) | 0.063  (-0.051, 0.176, p=0.28) | -0.0002  (-0.108, 0.107, p=0.99) |
| Male sex | 12.943 (10.187, 15.699, p<0.001) | 15.764  (12.293, 19.236, p<0.001) | 13.938  (10.885, 16.991, p<0.001) | 12.527  (9.636, 15.417, p<0.001) |
| BMI, kg/m^2^ | -0.297 (-0.515, -0.078, p=0.009) | -0.196  (-0.472, 0.079, p=0.16) | -0.069  (-0.311, 0.174, p=0.58) | 0.153  (-0.076, 0.382, p=0.19) |
| Ventilatory Ratio | -1.174 (-3.355, 1.008, p=0.29) | -0.710  (-3.458 , 2.037, p=0.61) | -1.015  (-3.432 , 1.401, p=0.41) | -0.659  (-2.946 , 1.629, p=0.57) |
| V_T_, ml⋅kg IBW | 3.987 (2.645, 5.330, p<0.001) | 4.828  (3.137, 6.519, p<0.001) | 4.754  (3.266, 6.241, p<0.001) | 3.980  (2.572, 5.388, p<0.001) |
| PaO_2_/FiO_2_ | 0.015 (-0.006, 0.037, p=0.16) | 0.019  (-0.008 , 0.046, p=0.17) | 0.013  (-0.010 , 0.037, p=0.27) | 0.016  (-0.007 , 0.038, p=0.17) |

A p value ≤ 0.05 was considered statistically significant.

Abbreviations: PEEP= positive end expiratory pressure, ARDS = acute respiratory distress syndrome, BMI= body mass index, V_T_,= tidal volume, IBW= ideal body weight, CI= confidence interval

**Table 7S. Results of the propensity score weighted association between C_rs,avg_ and COVID status at different PEEP levels, excluding extrapulmonary ARDS patients**

|  | **non-COVID ARDS** | **COVID ARDS** | **p value** |
| --- | --- | --- | --- |
| number of patients | 75 | 276 |  |
| C_rs,avg_  at PEEP 0 cmH_2_O | 37 [27, 47] | 33 [25, 42] | 0.009 |
| C_rs,avg_  at PEEP 5 cmH_2_O | 38 [28, 50] | 38 [28, 44] | 0.33 |
| C_rs,avg_  at PEEP 10 cmH_2_O | 36 [26, 49] | 36 [29, 44] | 0.85 |
| C_rs,avg_ at PEEP 15 cmH_2_O | 30 [21, 41] | 33 [24, 40] | 0.46 |

Data are shown as median [interquartile range]. A p value ≤ 0.05 was considered statistically significant.

Abbreviations: C_rs,avg_  = average respiratory system compliance, PEEP = positive end expiratory pressure, ARDS = acute respiratory distress syndrome
